# Pharmacists' perceptions about providing COVID-19 vaccines in community settings

**DOI:** 10.1016/j.rcsop.2023.100326

**Published:** 2023-09-05

**Authors:** Mansour M. Alotaibi, Eman M. Aldandan, Bashayer E. Alfredan, Samar H. Almohammed, Zahra H. Almousa

**Affiliations:** a Pharmacy Practice Department, College of Clinical Pharmacy, King Faisal University, Al-Ahsa 31982, Saudi Arabia; b Pharm-D intern, College of Clinical Pharmacy, King Faisal University, Al-Ahsa 31982, Saudi Arabia

**Keywords:** Community pharmacy, Pharmacists, Vaccinations, Vaccines, Immunizations, COVID-19, Coronavirus

## Abstract

**Background:**

Community pharmacists were underutilized in Saudi Arabia. At the start of the coronavirus disease 2019 (COVID-19) outbreak, global healthcare systems experienced significant pressure. To combat the pandemic effectively, there was a need to involve other healthcare providers, such as community pharmacists. As a result, community pharmacists were enlisted to provide vaccines.

**Objectives:**

This study aims to explore community pharmacists' perceptions about providing COVID-19 vaccines in Saudi community settings.

**Methods:**

Ethical approval was granted by the Research Ethics Committee of King Faisal University in January 2022. A qualitative methodology was used to explore the opinions of community pharmacists. Semi-structured interviews were conducted via face-to-face qualitative interviewing. Participants were recruited purposively and represented different types of community pharmacy settings.

**Results:**

A total of 15 face-to-face individual interviews were conducted. The cohort included pharmacists who provided the service (*n* = 5) and those who did not (*n* = 10). The results showed that providing the service in a community setting was advantageous to patients, community pharmacy sector, and healthcare system. Moreover, the participants identified several barriers to providing the service.

**Conclusion:**

Although providing the service had several advantages to several parties, the identified barriers need to be considered for the service to be provided appropriately.

## 1 Introduction

The Kingdom of Saudi Arabia is a Middle Eastern country located in Asia with a total area of approximately 2,000,000 square kilometers.^1^ It is a member of the Group of Twenty (G20) and one of the 20 largest economies in the world.^2^^,^^3^ The Kingdom had an estimated population of more than 34,000,000 as of 2021.^4^ The vision of the Kingdom of Saudi Arabia (vision 2030) was launched several years ago and gave rise to significant reforms at all levels, including the healthcare system.^5^^,^^6^

In Saudi Arabia, the traditional role of community pharmacists has mainly focused on the dispensing of medications and counselling of patients.^7^ As they are more accessible to patients than other healthcare facilities in Saudi Arabia, and because patients do not need to book an appointment to see a pharmacist, the community pharmacy sector was empowered to play an enhanced role in patient care.^8^^,^^9^ The sector has been evolving towards providing more patient centered care, including administering vaccines, measuring vital signs, training patients on how to use medical devices and health promotion.^9^

Since the outbreak of the coronavirus disease 2019 (COVID-19), the demand for healthcare services has increased, placing additional pressure on global healthcare systems.^10^ The COVID-19 outbreak negatively impacted healthcare systems around the world in terms of capacity, availability and quality of services provided.^11^^,^^12^ This affected not only hospitals and healthcare centers, but also community pharmacy settings. As a result, countries began to enact legislations to cope with the pandemic. In the Kingdom of Saudi Arabia, although there was an initiative to enable community pharmacists to provide flu vaccination services, this was not widely implemented. Therefore, the need arose for community pharmacists to take on non-traditional roles and provide additional clinical services. As a part of the adopted strategic plan of the Kingdom, a partnership agreement with large chain community pharmacies was made to provide citizens and residents with COVID-19 vaccines in a community pharmacy setting.^14^ This made the COVID-19 vaccines available at all healthcare sectors e.g. governmental and private hospitals, clinics, vaccination operation centers and community pharmacies.^14^ The vaccination service provided by all sectors was free to all people living in the country.^14^ In order to ensure the quality of the service, the Ministry of Health issued a blueprint which was used by the concerned institutions to initiate, provide and control the service.^14^ The Ministry of Health represented by the quality department was responsible to supervise and audit all activities related to providing the vaccines starting from supply chain to measuring customer experience.^14^ In addition to that, in-person/virtual training programs and educational courses were provided both at regional and site levels to ensure delivery of error-free immunization services.^14^

Notably, Saudi Arabia, according to the Ministry of Health, was at the top of the list of countries providing the COVID-19 vaccine. Vaccines that are currently available in Saudi Arabia include Pfizer-BioNTech, Oxford-AstraZeneca, Moderna and Janssen.^15^ Given that community pharmacists, as qualified healthcare providers, had a significant role in fighting COVID-19 by administering vaccines, there was a need to capture their perceptions about providing vaccination services in community pharmacies. Previous research conducted worldwide showed that there were several factors affecting provision of the service in community settings, such as, pharmacists knowledge and skills, community pharmacy resources, patient acceptability.^16^, ^17^, ^18^ A study conducted in Saudi Arabia indicated that pharmacists showed positive attitudes towards providing clinical services to patients.^19^ However, to the best of the authors' knowledge, this is the first qualitative study investigating community pharmacists' perceptions about providing Covid-19 vaccines in Saudi community settings.

### 1.1 Aim of the study

This study explores the perceptions of community pharmacists about providing COVID-19 vaccinations in Saudi community settings. The scope of the study was not only limited to the opinions of the community pharmacists who provided the vaccination services but also considered the feedback of those who did not.

## 2 Materials and methods

A qualitative methodology was used to explore the opinions of community pharmacists.^20^^,^^21^ The study was reported per COREQ criteria.^22^ Semi-structured interviews were conducted via face-to-face qualitative interviewing.^23^ Ethical approval was granted by the Research Ethics Committee of King Faisal University in January 2022. Participants were mainly recruited purposively to ensure the diversity of the cohort.^24^ Due to the limited number of community pharmacists who provided COVID-19 vaccination services, snowball sampling was also used.^25^ No personally identifiable information was disclosed as this might lead to identify participants. Moreover, the consent form completed by participants included a statement about protecting confidentiality of participants as they may discuss sensitive information. The researchers invited participants via face-to-face direct contact. Information sheets were provided to participants, and their consent was received prior to conducting the interviews. The topic guide was developed according to the literature and discussions with experts in the field.^26^ Open-ended questions were used to investigate the topic, allowing participants to express their opinions freely. The questions were constructed to understand the participants' viewpoints towards providing the COVID-19 vaccine in community pharmacy. To ensure the appropriateness and validity of the topic guide, a pilot study was conducted, including two interviews with community pharmacists.^21^^,^^27^ These two interviews were not included in the analysis.

### 2.1 Data collection

Data collection was conducted over two months (March and April 2022). Two audio recorders were used to record the interviews,^27^ all of which were conducted in Al-Ahsa governate, Saudi Arabia, at a location and time that suited the participants. Research team members (4 female PharmD interns) who had not previously conducted a qualitative study completed a training course, which was provided by an expert in qualitative methodology, and practiced conducting interviews before commencing the study. The course discussed important aspects of conducting qualitative interviews and dealing with qualitative data, such as transcribing, coding, and analysis. No relationship was established with participants prior to conducting the interviews, however; the interviewers asked general questions at the beginning of each interview to serve as icebreaker questions. Each interview lasted no more than 30 min and was transcribed verbatim and checked by the researchers,^27^ who also contacted participants for clarification when needed. Participants had the opportunity to check and approve all of the interviews. All interviews were translated into English and double-checked for accuracy by researchers fluent in both languages (i.e. Arabic and English languages). At the end of each interview, participants were asked if they wanted to add anything to the discussion to ensure that nothing was left undiscussed. The research team decided to cease data collection when saturation was reached.^28^ The primary researcher holds a PhD degree in pharmacy practice, practices as a community pharmacist, conducted several qualitative studies, and was actively involved in all the steps of research process.

### 2.2 Data analysis

Once the transcripts were ready for analysis, all personal identifiable information (e.g. participants and pharmacies names) were removed to ensure confidentiality. The transcripts were manually and independently coded by the research team. Continued meetings were conducted to ensure the appropriateness of the codes and themes generated. Inductive thematic analysis was used to identify themes within the data set.^29^^,^^30^ This would help in identifying barriers that face community pharmacists while providing the service. A six-stage-framework was implemented to analyze the date, as follows: familiarizing the researcher with the data, generating initials codes, creating themes, reviewing the themes, refining and defining the themes, and producing the report.

## 3 Results

A total of 15 community pharmacists agreed to take part in the study and were interviewed. The cohort sample included community pharmacists who provided the vaccination service (*n* = 5) and those who did not (*n* = 10). Moreover, 13 community pharmacists worked for large pharmacy chains and two worked at independent pharmacies. It is worth noting that some of those who did not provide the service were working in pharmacies that offered the service (*n* = 2), which may have enriched their knowledge of the investigated topic. As very few pharmacists were authorized to provide the service, and were included in the study, other personally identifiable information was not disclosed to protect participants confidentiality. Two main themes and several sub-themes emerged related to pharmacists' perceptions about providing COVID-19 vaccines in community settings (Table 1).

**Table 1:** The identified themes and subthemes.

1. **Benefits**
1.1 Benefits to patients
1.2 Benefits to community pharmacy
1.3 Benefits to healthcare system
2. **Barriers and Facilitators**
2.1 Barriers and facilitators related to patients
2.2 Barriers and facilitators related to community pharmacy
2.3 Barriers and facilitators related to policy and regulations

### 3.1 Benefits

Community pharmacists indicated that there were several advantages to providing COVID-19 vaccination services in community settings. The benefits were not only related to community pharmacies but also other stakeholders, such as patients and the healthcare system as a whole.

#### 3.1.1 Benefits to community pharmacy

Providing the service had a positive impact on community pharmacies in terms of their relationship with the public. People may gain more trust in community pharmacists who provide the service, leading to robust relationships between pharmacists and patients; and therefore, increasing pharmacies' income.

*‘I feel that providing the vaccination service in a community setting would enhance public trust in community pharmacies.’*

[*Chain community pharmacist 7, did not provide the service].*

*‘Providing the service will boost footfall in community pharmacies, and consequently the pharmacy's earnings would be increased as well.’*

[*Chain community pharmacist 10, did not provide the service].*

It was indicated that providing the service delivered personal satisfaction to community pharmacists.

*‘My experience in providing the vaccine was awesome. I felt that I had a crucial role in protecting the community from COVID-19 through providing the service.’*

[*Chain community pharmacist 4, provided the service].*

*‘I am grateful to God that I was able to perform the service and that people were well served. I mean, this is true inner satisfaction. Having been able to serve any customer or person in need is a pleasant feeling.’*

[*Chain community pharmacist 14, provided the service].*

#### 3.1.2 Benefits to patients

Participants expressed that providing vaccines in community settings would enhance the accessibility of the service, as community pharmacies are located almost everywhere, on main streets, in neighborhoods, cities and villages, and are typically open for longer working hours.

*‘As pharmacies are close to almost everyone, they can visit the pharmacy whenever they want to fulfil their health needs.’*

[*Chain community pharmacist 3, did not provide the service].*

‘Providing the service in community pharmacies is more convenient for patients, as they can receive the service easily and smoothly.’

[Chain community pharmacist 8, did not provide the service].

#### 3.1.3 Benefits to healthcare system

Participants claimed that healthcare facilities/providers would be able to deal with patients in need adequately as the pressure on healthcare services would be relieved.

*‘Providing the service in pharmacies reduces the pressure on hospitals and other vaccination facilities.’*

[*Chain community pharmacist 12, did not provide the service].*

### 3.2 Barriers and facilitators

Participants identified several challenges that could hinder the provisioning of vaccination services in community settings. These barriers are related to community pharmacy settings, patients and policies and regulations. To overcome these obstacles, participants noted several facilitators that could support providing the service.

#### 3.2.1 Barriers and facilitators related to community pharmacy

One of the major barriers to providing the service in community settings was related to workload and capacity. Adding new responsibilities to community pharmacists would make it difficult to perform their daily tasks.

*‘Pharmacists cannot perform all the tasks needed alone. There should be someone to help with data entry for example, so pharmacists can focus on providing the service.’*

[*Chain community pharmacist 7, did not provide the service].*

‘There were not enough pharmacists to handle all of the patients who showed up to receive the vaccines.’

[Chain community pharmacist 15, did not provide the service].

‘In each pharmacy, there must be a person whose responsibility is to check patients’ information and enter data.’

[Chain community pharmacist 13, provided the service].

As the number of beneficiaries increased in pharmacies providing the vaccination service, some may not have had sufficient space to accommodate customers.

‘A waiting area must be set up inside the pharmacy, so people can sit.’

[Chain community pharmacist 13, provided the service].

‘It is difficult to provide the service in here, as the pharmacy is small.’

[Independent community pharmacist 5, did not provide the service].

The number of people arriving to access vaccines in pharmacies was described as significant, and this may have impacted the quality of service provided and patient satisfaction.

‘The pharmacy was very busy. A lot of customers. One after another. A huge number. We vaccinated an enormous number of people every day.’

[Chain community pharmacist 15, provided the service].

‘Sometimes people coming to get the vaccine shot had to wait outside the pharmacy because we did not have enough space available.’

[Chain community pharmacist 6, provided the service].

One of the identified barriers was related to the availability of an adequate space in which to provide the service. Not all community pharmacies were equipped with consultation rooms in which patients could receive clinical services appropriately.

‘Our pharmacy is not adequate for offering the service, as it's small and there's no consultation room.’

[Independent community pharmacist 9, did not provide the service].

‘Not all pharmacies can have an adequate consultation room. However, if we were able to find a proper place in the pharmacy, such as a small room, or something similar, even if it was temporary, this would help the process along.’

[Chain community pharmacist 7, did not provide the service].

One pharmacist went beyond having proper consultation rooms and suggested that pharmacies should have proper emergency service/ambulance services ready to be used when needed.

‘One thing that needs to be considered is the availability of emergency services; however, we have not needed such a service, but this may be needed in some cases.’

[Chain community pharmacist 1, provided the service].

Participants indicated that vaccines required proper storage procedures that may not have been available in all community pharmacies. Not following the correct procedures could affect the vaccines' safety and effectiveness.

‘The refrigerators should have certain specifications, and pharmacy staff should know how to properly store vaccines.’

[Chain community pharmacist 13, provided the service].

Another barrier that was noted by participants was related to the competency of community pharmacists to provide the service. Provision of the service required qualified healthcare providers. Given that most community pharmacists in Saudi Arabia had not been trained to provide the service, this may have impacted the availability of the service in community settings.

‘This is due to the fact that the vaccine must be administered by a qualified person who has been properly trained on how to do so.’

]Chain community pharmacist 4, provided the service].

‘We have been trained intensively on how to administer vaccines, how to handle the vaccine itself, and how to store it properly.’

[Chain community pharmacist 13, provided the service].

#### 3.2.2 Barriers and facilitators related to patients

Some participants indicated that people might not be willing to get vaccines in community pharmacies, as they were not nationally well known for providing such a service.

*‘One of the facilitators that helped us a lot was related to the fact that we have been providing the flu vaccine in this pharmacy for a long time, so people trust us as vaccine providers.’*

[Chain community pharmacist 14, provided the service].

Although there was a booking system in place for people to get the service through community settings, the participants claimed that some people did not adhere to the policy regulating the service provision (e.g. approaching pharmacies to get the service without booking an appointment). Others may have been unfamiliar with the availability of the service in a community setting.

‘People often attempt to obtain vaccinations without scheduling an appointment.’

]Chain community pharmacist 6, provided the service].

‘It is important for each person to keep to their appointment, so that the pharmacist can provide the service at their own pace without crowding.’

[Chain community pharmacist 2, did not provided the service].

‘You must put stickers, a large sticker here to inform the public that there is a vaccination center inside the pharmacy.’

[Chain community pharmacist 13, provided the service].

‘I was working at a pharmacy that's close to another one that offered the service. People were often approaching me and asking for the service. There should be a way to inform people about pharmacies that offer the service.’

[Chain community pharmacist 11, did not provide the service].

#### 3.2.3 Barriers and facilitators related to policy and regulations

Several participants claimed that the service was not adequately rewarded. They suggested that good financial incentives should be offered to compensate pharmacists and encourage them to provide the service.

*‘Obviously, it is better if there is a financial incentive. We are not going to say no, but we should fulfil our obligations. All of us earn a salary and are aware of our financial position. It is not affected, thank God. But of course, if there is a financial incentive, why not? Nobody will hate that.’*

[*Chain community pharmacist 14, provided the service].*

*‘The financial incentive could be a good thing. Offering a financial incentive will make it easier for me to deal with the heavy workload and long working hours. All of my pharmacy colleagues felt overstretched when providing this service. So, if there is a financial incitive, that is fine, but for now there is nothing more than that it is a community service, I mean, I'm sure I wish to serve the community, but I don't know...’*

[*Chain community pharmacist 15, did not provide the service].*

*‘It is good to serve our community, but we are not a charity! We are working for a for-profit organization, so everyone here expects to get paid for any additional task assigned. Providing the COVID-19 vaccine was a very difficult job, too many people coming for the vaccine, and there was no break.’*

[*Chain community pharmacist 15, did not provide the service].*

One participant seemed partially satisfied with the financial incentive provided by his/her organization, as the remuneration model was shifted from the number of products sold to the number of people vaccinated.

*‘My commission was calculated based on the number of vaccinated people instead of a sales target, so there was no significant drop in my earnings.’*

[*Chain community pharmacist 14, provided the service].*

Participants reported that they experienced difficulties providing the service due to issues related to responsible parties, such as a shortage of vaccines, medical supplies and the availability of appointments.

*‘Sometimes we faced vaccine shortages or delays related to medical supplies.’*

[*Chain community pharmacist 14, provided the service].*

*‘One of the barriers we encountered was related to the booking system that's managed by the Ministry of Health. Sometimes they allowed for people to book, sometimes not.’*

[*Chain community pharmacist 14, provided the service].*

The availability of appointments was mainly dependent on the availability of vaccines. The Ministry of Health offered vaccination appointments at all vaccination centers via an electronic booking system, but this could only be done when the vaccines were available.

## 4 Discussion

Community pharmacists are healthcare providers that are not effectively utilized.^31^^,^^32^ Their role is mainly focused on medication dispensing.^7^ Given that the majority of Saudi pharmacy colleges offer doctor of pharmacy programs (Pharm-D),^33^ pharmacists should take on an additional role in patient-focused care. The outbreak of COVID-19 has placed huge pressure on healthcare systems.^34^ Not having an adequate number of healthcare providers due to the increased demand for clinical services, and healthcare providers being absent because of COVID-19, were all factors that contributed to involving community pharmacists in the provisioning of clinical services, such as immunization. However, the findings of this study showed that several barriers arose for pharmacies providing COVID-19 vaccines. These barriers were not limited to community pharmacists but also affected other stakeholders, such as patients and policymakers.

In Saudi Arabia, the doctor of pharmacy students are not trained to administer vaccines as it is not part of the curriculum or their training.^35^^,^^36^ This means that pharmacists currently practicing in Saudi Arabia require further training to master vaccine administration. Additionally, the vast majority of community pharmacists practicing in the Kingdom are not Saudis^37^; they hail from different countries and studied at different pharmacy schools. The findings of this study are in line with previous research conducted elsewhere that have shown that community pharmacists needed additional training and educational courses to ensure the quality of the vaccination service.^16^^,^^17^ Therefore, assurances should be made that those who provide vaccination services are competent to ensure best quality of service provision. Another barrier that arose, which the majority of participants indicated, was that community pharmacies were not properly equipped to provide the vaccination service. These findings corroborate with previous research that indicated the need of consultation rooms and support in community pharmacy settings.^16^^,^^19^ Community pharmacies must invest more in establishing consultation rooms, as most clinical services should be provided in an environment that maintains patient privacy. Furthermore, since certain COVID-19 vaccines require strict specifications for storage and handling, this may limit the ability of community pharmacies to offer all of the available vaccines on the market.

This study showed that certain policies that regulate the community pharmacy sector in Saudi Arabia, at national and organizational levels, may hinder the provision of the service in community settings. Participants indicated that community pharmacists may not be properly rewarded for providing vaccination services. To provide high-quality patient care, a remuneration model that financially rewards healthcare providers should be put in place. Pharmacists' remuneration for COVID-19 vaccinations are varied worldwide. Community pharmacies/Pharmacists practicing in Australia, Switzerland, UK, and US receive $10.29 – $40 per booster vaccine administered.^38^, ^39^, 40., 41 The most important factor that should be considered when it comes to remuneration is paying pharmacists per each vaccine administered. The remuneration should also consider supplies needed, the vaccine acquisition cost if is not purchased by the Government. Another point that was highlighted related to the availability of the vaccination service. As the agreement of providing immunization inside community pharmacies was made with large pharmaceutical chains,^13^ it may limit the involvement of other community pharmacies and, consequently, their ability to serve the community. This may have been reasonable when the immunization program was launched in Saudi Arabia, due to the limited amount of vaccines available at the time. However, subsequently, all community pharmacies should have been given an equal opportunity to provide this service, as long as they met the service specifications.

Saudi Arabia was one of the first countries to provide COVID-19 vaccines free of charge to all its residents (i.e. Saudis and non-Saudis).^42^ A significant advertising drive took place throughout the Kingdom to increase awareness of the importance of COVID-19 vaccines and how to be immunized. The service was available through the Sehhaty application, developed by the Saudi Ministry of Health.^43^^,^^44^ Residents could access the application and select where to receive the service and the specific vaccine they preferred. Although the booking process for vaccines was simple and convenient,^45^ the findings of this study showed that some people did not follow the appropriate steps for being vaccinated. Some approached community pharmacists without booking appointments. This increased the workload on pharmacists and may have impacted the experience of other customers who had appointments. Moreover, one of the identified barriers related to patients, was their willingness to be vaccinated in a community setting. People may not have wanted to be vaccinated with the COVID-19 vaccine in a community setting. The same finding was observed in previous research which has shown that people had negative opinions towards receiving vaccines in a community setting.^46^ The intake of community pharmacies could be increased by emphasizing pharmacists' competency and expertise (e.g. being certified to provide the service), having proper locations such as consultation rooms and focusing on medically fit customers (e.g. customers with no known allergies).

This was the first study to explore community pharmacists' views about providing COVID-19 vaccines in Saudi community pharmacy settings. Although participants were recruited from Al-Ahsa community pharmacies, the purposive sampling reduced the impact of this limitation. Several participants were recruited from the largest Saudi community pharmacy chains that have branches in Al-Ahsa governate. Furthermore, the selected participants included pharmacists who provided the service and those who did not. All of these factors may have enhanced the representation of the sample. Finally, not providing personally identifiable information for participants (age, gender, etc.) might be a limitation of this study; however, a decision was made to not include them as very few pharmacists were authorized to provide the service in Al-Ahsa governate which may threaten participant confidentiality.

## 5 Conclusions

Involving community pharmacists in providing COVID-19 vaccines might be beneficial to the community pharmacy sector, patients and the healthcare system as a whole. However, several barriers must be properly addressed to enable the provisioning of a seamless and high-quality service. Future research that assess individuals acceptability to receive vaccines at a community pharmacy setting may provide better insight. Understanding the factors that determine the patient preference may lead to improve vaccination service uptake in community pharmacies. Assessing the capability of pharmacists to provide the service via conducting a quantitative approach might also lead to better understanding of the topic.

## Funding

This research was funded by King Faisal University, Saudi Arabia, grant number 630

## Declaration of Competing Interest

The authors declare no conflict of interest.

## Appendix A Supplementary data

Supplementary material Image 1
